# *Trypanosoma cruzi* Intracellular Amastigotes Isolated by Nitrogen Decompression Are Capable of Endocytosis and Cargo Storage in Reservosomes

**DOI:** 10.1371/journal.pone.0130165

**Published:** 2015-06-09

**Authors:** Cassiano Martin Batista, Rafael Luis Kessler, Iriane Eger, Maurilio José Soares

**Affiliations:** 1 Laboratório de Biologia Celular, Instituto Carlos Chagas/Fiocruz-PR, Curitiba, Paraná, Brazil; 2 Laboratório de Genômica Funcional, Instituto Carlos Chagas/Fiocruz-PR, Curitiba, Paraná, Brazil; 3 Departamento de Biologia Geral, Universidade Estadual de Ponta Grossa, Ponta Grossa, Paraná, Brazil; University of Sao Paulo, BRAZIL

## Abstract

Epimastigote forms of *Trypanosoma cruzi* (the etiologic agent of Chagas disease) internalize and store extracellular macromolecules in lysosome-related organelles (LROs) called reservosomes, which are positive for the cysteine protease cruzipain. Despite the importance of endocytosis for cell proliferation, macromolecule internalization remains poorly understood in the most clinically relevant proliferative form, the intracellular amastigotes found in mammalian hosts. The main obstacle was the lack of a simple method to isolate viable intracellular amastigotes from host cells. In this work we describe the fast and efficient isolation of viable intracellular amastigotes by nitrogen decompression (cavitation), which allowed the analysis of amastigote endocytosis, with direct visualization of internalized cargo inside the cells. The method routinely yielded 5x10^7^ amastigotes—with typical shape and positive for the amastigote marker Ssp4—from 5x10^6^ infected Vero cells (48h post-infection). We could visualize the endocytosis of fluorescently-labeled transferrin and albumin by isolated intracellular amastigotes using immunofluorescence microscopy; however, only transferrin endocytosis was detected by flow cytometry (and was also analyzed by western blotting), suggesting that amastigotes internalized relatively low levels of albumin. Transferrin binding to the surface of amastigotes (at 4°C) and its uptake (at 37°C) were confirmed by binding dissociation assays using acetic acid. Importantly, both transferrin and albumin co-localized with cruzipain in amastigote LROs. Our data show that isolated *T*. *cruzi* intracellular amastigotes actively ingest macromolecules from the environment and store them in cruzipain-positive LROs functionally related to epimastigote reservosomes.

## Introduction

The internalization of extracellular macromolecules by eukaryotic cells occurs by clathrin-mediated or clathrin-independent endocytosis [1–4]. In the protozoan parasite *Trypanosoma cruzi* (Euglenozoa: Kinetoplastea), a hemoflagellate that causes Chagas disease in humans [5–8], endocytic events are well characterized in epimastigotes, proliferative forms found in the insect vector.

*T*. *cruzi* epimastigotes ingest macromolecules via endocytic vesicles formed at two specialized cortical structures located at the anterior of the cell: the cytostome/cytopharynx complex and the flagellar pocket membrane [5, 6, 9]. After endocytosis, internalized macromolecules are directed to lysosome-related structures (LROs) called reservosomes, and co-localize with cruzipain, the major cysteine proteinase in *T*. *cruzi* [5, 10–12]. All *T*. *cruzi* developmental forms have LROs, as shown by the co-localization of serine carboxypeptidase, cruzipain and chagasin in axenic epimastigotes, intracellular and tissue culture-derived amastigotes, and trypomastigotes obtained from culture supernatants [13]. However, contrary to the role of reservosomes in epimastigotes, it is possible that the LROs of intracellular amastigotes may not be used for the storage of extracellular macromolecules internalized by the parasite [13].

The iron transporter transferrin [14] is a key molecule internalized by *T*. *cruzi* epimastigotes. In trypanosomatids, transferrin obtained by endocytosis is the main source of iron ions that are essential for DNA replication, antioxidant defense, mitochondrial respiration and also for the synthesis of the modified base ‘J’ [15]. Previous works indicate that transferrin uptake in epimastigotes occurs by receptor-mediated (but clathrin-independent) endocytosis, mainly through the cytostome/cytopharynx [6,16], while albumin internalization occurs by clathrin-dependent endocytosis at the flagellar pocket membrane [6–8].

While endocytosis by *T*. *cruzi* epimastigotes has been studied in detail, the endocytic activity of proliferative intracellular amastigotes—the most clinically relevant form of the parasite—is poorly understood. Morphologically, the presence of a cytostome [17] and of LROs with large electron-lucent rods [13] (similar to the reservosomes found in epimastigotes [8,18]) suggest that endocytosis is likely to occur in the amastigote form. However, endocytosis has rarely been detected in intracellular *T*. *cruzi* amastigotes.

A pioneering study in 1973 showed that intracellular *T*. *cruzi* “spheromastigotes” (i.e., amastigote-like forms) incorporated melanin granules from chick embryo pigmented epithelial cells, via the cytostome [19]. Also, *T*. *cruzi* amastigotes express receptors for human transferrin [20], and require iron for growth in axenic conditions [21], in peritoneal macrophages *in vitro*, and in mice [22]. Amastigotes internalize holo-transferrin (i.e., iron-loaded transferrin), as shown by the inability of acid treatment to dissociate amastigote-bound holo-transferrin at 37°C, when endocytosis is active, while dissociation occurs readily at 4°C, when endocytosis is blocked [20]. However, these authors could not demonstrate the intracellular localization of the internalized transferrin. More recently, Waghabi and co-workers (2005) showed that intracellular amastigotes internalize transforming growth factor-β (TGF-β) from host cardiomyocytes (by a poorly characterized process of receptor-mediated endocytosis), and that TGF-β uptake is important to regulate the intracellular life cycle of the parasite [23]. Nevertheless, the exact localization of the internalized TGF-β could not be determined. Finally, an ultrastructural study showed a few surface-bound transferrin-gold complexes in amastigotes collected from the supernatant of infected Vero cell cultures, but again no intracellular labeling was found [5].

The difficulty in detecting endocytosis in intracellular amastigotes is likely due to the fact that endocytic assays are technically challenging in this life cycle form. When assays are performed using infected cells, labeled tracers are first taken up by the host cells and, therefore, are mostly unavailable for uptake by intracellular amastigotes in the host cell cytoplasm [24]. Performing endocytosis assays with isolated amastigotes may represent an interesting alternative. Intracellular amastigotes can be isolated directly from infected spleen and liver [25] or from infected tissue culture cells [26, 27] by density gradient centrifugation (using metrizamide and/or Percoll). Density gradient-based protocols are, however, very laborious and involve several medium changes, which can lead to a considerable loss of parasite viability. A different protocol, adapted from a method to purify *T*. *cruzi* metacyclic trypomastigotes [28, 29], used anion-exchange chromatography to separate *T*. *cruzi* intracellular amastigotes from cell debris and trypomastigotes, after needle-based disruption of infected cells [30]. Although this is a robust option to purify amastigotes in a larger scale, chromatography is also time consuming, and the positively-charged resin may induce changes in the parasite’s surface glycoconjugates.

In the present work, we introduce a rapid cavitation procedure to isolate *T*. *cruzi* amastigotes from Vero cells. In this protocol, cavitation by nitrogen decompression is followed by a few centrifugation steps (but no density gradients), allowing the rapid purification of viable intracellular amastigotes that are competent for endocytosis. Flow cytometry, fluorescence microscopy and western blotting analyses demonstrated that the *T*. *cruzi* amastigotes isolated using this new methodology were capable of internalizing transferrin and albumin from the extracellular milieu, and that these molecules were directed efficiently to LROs. Importantly, we detected co-localization of ingested transferrin and albumin with cruzipain, in LROs. Our data also suggest that the LROs of *T*. *cruzi* intracellular amastigotes correspond to reservosomes.

## Materials and Methods

### Reagents

BCIP-NBT Color Development Substrate was purchased from Promega Corporation (Madison, WI, USA). Alkaline phophatase (AP)-conjugated rabbit anti-goat IgG and AP-conjugated rabbit anti-mouse IgG were purchased from Santa Cruz Biotechnology (Santa Cruz, CA, USA). Holo-transferrin, bovine serum albumin, Roswell Park Memorial Institute-1640 (RPMI-1640) medium, Dulbecco’s modified Eagle medium (DMEM) and anti-transferrin goat IgG were purchased from Sigma-Aldrich (St. Louis, MO, USA). Bench Mark Protein Ladder, albumin-Alexa 488, transferrin-Alexa 633, Hoechst 33342, goat anti-mouse antibody coupled to AlexaFluor 488 or 594, and rabbit anti-goat antibody coupled to AlexaFluor 594 were purchased from Invitrogen-Life Technologies (Carlsbad, CA, USA). Prolong Gold antifade reagent was purchased from Molecular Probes-Life Technologies (Eugene, OR, USA). Fetal bovine serum (FBS) was purchased from Cultilab Ltda (Campinas, SP, Brazil). The anti-Ssp4 2C2 monoclonal antibody [30] was kindly provided by Dr. Renato Mortara (UNIFESP, Brazil).

### Parasites

Culture epimastigote forms of *Trypanosoma cruzi* clone Dm28c [31] were maintained by weekly passages in liver infusion tryptose (LIT) medium [32] supplemented with 10% heat-inactivated fetal bovine serum (FBS), at 28°C.

*In vitro*-derived *T*. *cruzi* metacyclic trypomastigotes were obtained by incubating epimastigotes in TAU3AAG medium, according to a previously described metacyclogenesis (i.e., epimastigote-to-trypomastigote differentiation) protocol [33]. After 72 h of cultivation in this medium, ~80% of the cells in the supernatant were trypomastigotes.

Vero cells (ATCC CCL-81) were maintained in 75-cm^2^ cell culture flasks (Corning Incorporated, Corning, NY, USA), in RPMI medium supplemented with 5% FBS, at 37°C (in a humidified 5% CO_2_ atmosphere). Cells were infected with *in vitro*-derived metacyclic trypomastigotes (at a ratio of 10 parasites/cell), 24h after passage. After 4 h of interaction, host cell monolayers were washed with PBS (to remove non-adherent parasites) and then incubated in the same conditions with 7–10 ml DMEM medium supplemented with 10% FBS for four days, when trypomastigote production peaked. Then, culture supernatants were collected, and cell-derived trypomastigotes that had been released into the supernatant were harvested by centrifugation at 3,000g, for 15 min.

Axenic amastigotes were obtained by *in vitro* amastigogenesis [34]. Briefly, trypomastigotes were collected from the supernatant of infected Vero cell cultures (4-days post-infection), washed with PBS and incubated in high glucose DMEM medium, at pH 5, and 37°C (in a humidified 5% CO_2_ atmosphere). After 24 hours, almost 100% of the cells had an amastigote shape.

### Isolation of *T*. *cruzi* intracellular amastigotes by nitrogen cavitation

To isolate intracellular amastigotes from host cells, five culture flasks with 1x10^6^ infected Vero cells each were washed three times with PBS (under gentle agitation), 48 hours post-infection (i.e., at the peak of intracellular amastigote replication), to eliminate contamination with extracellular amastigotes. Then, cultures were trypsinized for 10 min at 37°C, and the trypsinization was halted by the addition of cold FBS 1:1 (v/v). The infected cells in suspension were lysed by nitrogen decompression (cavitation), in a 4639 cell disruption vessel (Parr Instrument Company, Moline, IL, USA), using 180 psi pressure, for 5 min. Intact Vero cells were removed by centrifugation at low speed (10 min, 800*g*), and intracellular amastigotes were recovered from the supernatant. Cell debris were removed from the amastigote fraction by three centrifugation steps at 2,000*g* for 5 minutes, to produce a supernatant of isolated intracellular amastigotes.

### Endocytosis assay

Isolated intracellular amastigotes, axenic amastigotes and culture epimastigotes (positive control) were subjected to a previously described endocytosis assay [5], using 2x10^6^ parasites for flow cytometry analyzes, 5x10^6^ for fluorescence microscopy studies and 10^7^ for western blotting. Briefly, after 15 min under stress in PBS at 25°C, parasites were incubated with 50 μg/ml transferrin-AlexaFluor 633 or albumin-AlexaFluor 488, for 30 min, at 37°C (amastigotes) or 28°C (epimastigotes). Alternatively, parasites were incubated at 4°C to allow transferrin binding without internalization [6], or the retention of albumin in the flagellar pocket [10]. Negative control cells were incubated in the absence of labeled transferrin or albumin.

### Transferrin dissociation assay

Membrane-bound transferrin was dissociated using a previously described method [17], with modifications. Parasites were subjected to endocytosis assays as described above (using 50 μg/ml transferrin-AlexaFluor 633) and then incubated for 5s at 4°C with 0.25 M acetic acid/0.5 M sodium chloride, pH 3.5, after which the pH was quickly neutralized with 1 M sodium acetate. Transferrin dissociation from the parasite’s surface was analyzed by western blotting and flow cytometry, as described below.

#### Western blotting

For western blotting, 10^7^ epimastigotes or isolated intracellular amastigotes that had been subjected to endocytosis and transferrin dissociation assays (using 125 μg/ml transferrin-AlexaFluor 633) were washed, ressuspended in 15 μL PBS + 5 μL denaturing buffer (final concentration: 40 mM Tris-HCl pH 6.8, 1% SDS, 2.5% β-mercaptoethanol, 6% glycerol and 0.005% bromophenol blue), boiled for 5 min and then vortexed. Samples were run on 10% SDS-PAGE gels, and then transferred (by wet transfer) onto nitrocellulose membranes (Hybond C, Amersham Biosciences, Buckinghamshire, United Kingdom) according to standard protocols [35]. Membranes were blocked in blocking buffer (5% non-fat milk/0.05% Tween-20 in PBS), which was also used for all antibody incubations. After blocking, membranes were incubated for 2 h with an anti-transferrin goat antiserum (1:200, dilution), and then for 1 h with a mouse antiserum against TcGAPDH (1:5000 dilution) [36]. After 3 washes with 0.05% Tween-20/PBS, membranes were incubated for 1h with AP-conjugated rabbit anti-goat (1:7000 dilution) and AP-conjugated rabbit anti-mouse (1:500 dilution) IgG antibodies, washed 3 times with 0.05% Tween 20/PBS, and reactive bands were visualized using the BCIP-NBT solution, according to the manufacturer’s instructions. The Bench Mark Protein Ladder (10–220 kDa) was used to determine molecular weights.

Transferrin band densitometry was performed using the ImageJ 1.45s software (National Institute of Mental Health-NIMH, Bethesda, Maryland, USA). Integrated band densities estimated by ImageJ were used to calculate the ratios between the signal at 28°C (epimastigotes) or 37°C (amastigotes) and that at 4°C, for both untreated and acetic acid-treated parasites.

### Flow cytometry

Live parasites that had internalized transferrin-AlexaFluor 633 or albumin-AlexaFluor 488 were analyzed in a FACSAria II flow cytometer (Becton-Dickinson, San Jose, USA), using 660/20 and 530/30 nm band-pass filters, respectively. A total of 20,000 events were acquired in the regions of the FSC x SSC plot previously shown to correspond to parasites [37]. Data analysis was performed using the FlowJo software (FlowJo, Ashland, OR, USA), and the normalized median fluorescence intensities of transferrin and albumin were calculated as the ratios between the median fluorescence intensities of treated and untreated cells. Statistical analysis was performed using the software GraphPad Instat (GraphPad Software Inc, La Jolla, CA, USA), by ANOVA followed by Tukey-Kramer multiple comparisons test.

To detect the amastigote-specific surface protein Ssp4 [38], *in vitro*-derived metacyclic trypomatigotes and amastigotes (intracellular and axenic) were washed twice in PBS and fixed for 15 min in 4% formaldehyde. Cells were then incubated for 30 min at 28°C with an anti-Ssp4 monoclonal antibody [38] diluted to 1:3200, in PBS (pH 7.2). Samples were washed with PBS and incubated for 30 min at 28°C with a goat anti-mouse secondary antibody coupled to AlexaFluor 488 (1:1000 in PBS). Cells were washed with PBS and immediately analyzed by flow cytometry (as described above), using a 530/30 nm band pass filter. In parallel, anti-Ssp4 stained cells were adhered to poly-L-lysine coated slides and observed under a Nikon E600 epifluorescence microscope (Nikon Instruments, Tokyo, Japan).

### Immunofluorescence microscopy

To detect native cruzipain, isolated amastigotes and culture epimastigotes were washed twice in PBS, fixed for 30 min with 4% paraformaldehyde, permeabilized for 5 min in 0.5% Triton in PBS, and incubated for 2 h at 37°C with 50 μg/ml anti-cruzipain monoclonal antibody (CZP-315.D9; [39]), in PBS. Samples were then washed three times in PBS and incubated, for 2 h at 37°C, with a goat anti-mouse secondary antibody coupled to AlexaFluor 488 diluted to 1:600 in ‘incubation buffer’ (PBS with 1.5% BSA).

To co-localize transferrin-AlexaFluor 633 or albumin-AlexaFluor 488 with cruzipain, parasites that had been subjected to endocytosis assays were fixed for 30 min in 4% paraformaldehyde and permeabilized for 5 min with 0.5% Triton in PBS. For co-localization with transferrin-AlexaFluor 633, samples were incubated with both CZP-315.D9 (50 μg/ml) and the anti-transferrin goat antiserum (to enhance the AlexaFluor 633 signal; diluted to 1:150 in incubation buffer), washed three times in PBS and then incubated with goat anti-mouse-AlexaFluor 488 (to detect cruzipain) and rabbit anti-goat—AlexaFluor 594 (to detect transferrin) secondary antibodies (diluted to 1:600 in incubation buffer). For co-localization with albumin-AlexaFluor 488, samples were incubated with CZP-315.D9 and then with a goat anti-mouse-AlexaFluor 594 secondary antibody (using the same dilution conditions mentioned above).

After antibody incubations, all samples were washed three times in PBS, incubated for 5 min with 1.3 nM Hoechst 33342, mounted with Prolong Gold antifade reagent and examined under a Nikon Eclipse E600 epifluorescence microscope.

## Results and Discussion

### Rapid and efficient isolation of viable *T*. *cruzi* intracellular amastigotes by nitrogen decompression

*T*. *cruzi* epimastigote forms internalize albumin and transferrin and store these molecules in reservosomes, the late endocytic organelles in this parasite stage [5,8]. In contrast, little is known about the endocytic activity of the proliferative intracellular amastigote form, which prevails in chronic chagasic patients and represents the most clinically relevant form of this parasite [40]. Thus, the aim of our study was to analyze the endocytic activity of amastigotes by using transferrin and albumin as probes.

Given that the study of endocytosis by intracellular amastigotes is hindered by the concomitant endocytic activity of host cells, we first attempted to make fluorescent-labeled endocytic tracers available to amastigotes directly in the host cell’s cytoplasm, using mild detergent treatments (with saponin and lysolecithin) of infected cells; however, host cells were killed by the lowest detergent concentrations required for transferrin entry into the cytoplasm (data not shown).

Therefore, we developed a protocol to isolate viable, endocytosis-competent intracellular amastigotes from infected host cells, using nitrogen decompression (also known as nitrogen cavitation). Nitrogen decompression is an effective method for lysis and homogenization of cell suspensions [41], and is based on oxygen-free nitrogen dissolution under high pressure. After a sudden release of the pressure, nitrogen ‘bubbles out’ from the solution, including the cell interior, culminating in plasma membrane disruption. Depending on the cell type, higher pressures (leading to higher nitrogen dissolution) are needed for effective lysis. Mammalian cells are successfully disrupted after the release of 250–500 psi of pressure, while smaller cells with a resistant cell wall, such as bacteria, need around 1,500 psi [41]. Trypanosomatids have a sub-pellicular corset of microtubules that maintains parasite shape and provides resistance to mechanical pressure [42]. Hence, higher pressures are required to disrupt *T*. *cruzi* cells by cavitation, when compared with those needed to lyse mammalian cells, and we explored this difference efficiently to release intracellular amastigotes from infected cells.

**Fig 1:**
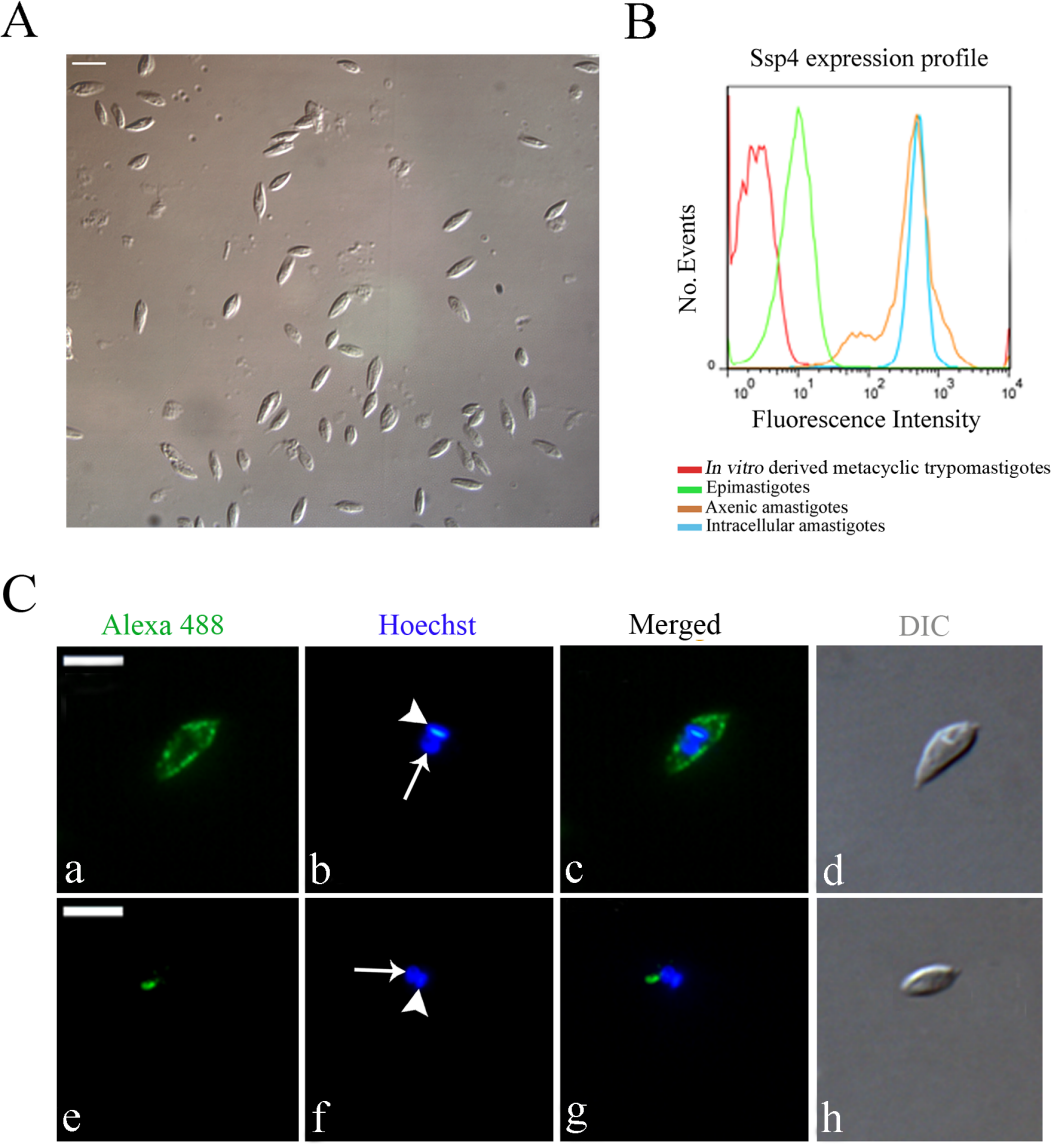
*Trypanosoma cruzi* intracellular amastigotes isolated by nitrogen decompression have normal shape, high levels of Ssp4, and cruzipain labeling in posterior organelles. (A) Differential interference contrast (DIC) image of the fraction of isolated intracellular amastigotes obtained after nitrogen decompression and differential centrifugation. Note that most cells have the typical amastigote shape. Scale bar, 10 μm. (B) Flow cytometry analysis of axenic and isolated intracellular amastigotes, culture epimastigotes and *in vitro*-derived trypomastigotes labeled with an antibody against Ssp4, a specific amastigote marker. Trypomastigotes and epimastigotes had low levels of fluorescence signal (possibly due to auto-fluorescence), while axenic and intracellular amastigotes showed higher fluorescence intensity. (C) Immunofluorescence microscopy of isolated intracellular amastigotes labeled with an anti-Ssp4 antiserum (green, in a-d), or with the anti-cruzipain monoclonal antibody CZP-315.D9 (green, in e-h). The nucleus (arrow) and the kinetoplast (arrowhead) are stained blue with Hoechst 33342, and parasite morphology was visualized by DIC. While Ssp4 localizes to the cell surface, the cruzipain signal is found specifically in lysosome related organelles posterior to the nucleus. Scale bars, 5 μm.

Cell disruption by nitrogen decompression allowed the release of ~5x10^7^ amastigotes per 5x10^6^ infected Vero cells (NIH 3T3 fibroblasts and H9C2 myoblasts were also used successfully for intracellular amastigote isolation; data not shown). Morphological analysis by light microscopy showed that the parasites obtained by nitrogen cavitation had the typical amastigote shape (Fig 1A). Flow cytometry analysis of parasites labeled for the amastigote-specific surface protein Ssp4 showed that isolated intracellular amastigotes had strong Ssp4 expression, similar to that observed in axenic amastigotes (Fig 1B), while trypomastigotes and epimastigotes did not express Ssp4. These data confirmed that the isolated parasites were indeed amastigotes. Interestingly, isolated intracellular amastigotes had a narrower profile of Ssp4 expression than axenic amastigotes, indicating that axenic amastigotes constitute a more heterogeneous population formed by parasites in a variety of differentiation stages.

Infected Vero cells containing intracellular amastigotes were more susceptible to cavitation than uninfected ones, and could be lysed effectively with as little as 180 psi, a condition that did not affect intracellular amastigotes (S1 Fig). Flow cytometry using a GFP-fluorescent *T*. *cruzi* clone [43] showed that, after cavitation, almost all infected Vero cells were disrupted, releasing GFP-positive viable intracellular amastigotes (S1 Fig). In contrast, uninfected Vero cells could only be lysed efficiently after the release of 350–400 psi, which also disrupted ~25% of intracellular amastigotes (not shown). Hence, by performing nitrogen cavitation at 180 psi, only infected cells were disrupted, resulting in increased recovery of intact amastigotes and a reduction in the amount of cell debris, which could be removed easily by successive washes (Fig 1A). We used a 5-min cavitation period routinely, and longer cavitation periods (10 min) did not increase parasite yield (data not shown).

We confirmed the expression of Ssp4 by indirect immunofluorescence, and also to analyzed the localization of the reservosome/LRO marker cruzipain in intracellular amastigotes (Fig 1C). We observed a strong Ssp4 signal on the cell surface of isolated intracellular amastigotes (Fig 1C), as previously described for extracellular amastigotes obtained from the transformation of blood or cell culture trypomastigotes [38]. The anti-cruzipain monoclonal antibody CZP-315.D9 recognized an LRO-like structure posterior to the nucleus (Fig 1C), a localization compatible with that of LROs [13].

Previously described methods for the isolation of *T*. *cruzi* amastigotes are laborious and involve several medium changes and/or anion-exchange chromatography [25–29], with considerable loss of parasite viability and likely changes in the amastigote surface glycoconjugates (due to the interaction with the positively-charged resin). The nitrogen decompression method for intracellular amastigote isolation described here is easier and faster than previously described protocols, and yields viable amastigotes efficiently, and is likely to represent an excellent tool for studies on different aspects of amastigote biology.

### Isolated intracellular amastigotes internalize fluorescently-labeled transferrin

Most data suggestive of endocytosis by amastigotes is indirect [19–23]. Soares and De Souza (1991) showed direct evidence of transferrin binding to the surface of cell-derived amastigotes, but not of transferrin internalization, suggesting a low level of endocytic activity by amastigotes [5]. In our work we have also tried to visualize transferrin endocytosis directly by transmission electron microscopy (TEM) in isolated intracellular amastigotes, but no intracellular transferrin-gold complexes were observed (data not shown). These results were likely due to the relatively low endocytic activity of amastigotes, compared with that of epimastigotes, where cargo-gold complexes can be readily detected by TEM. Thus, we attempted to detect endocytosis in isolated intracellular amastigotes by more sensitive techniques than TEM, such as western blotting and flow cytometry.

**Fig 2:**
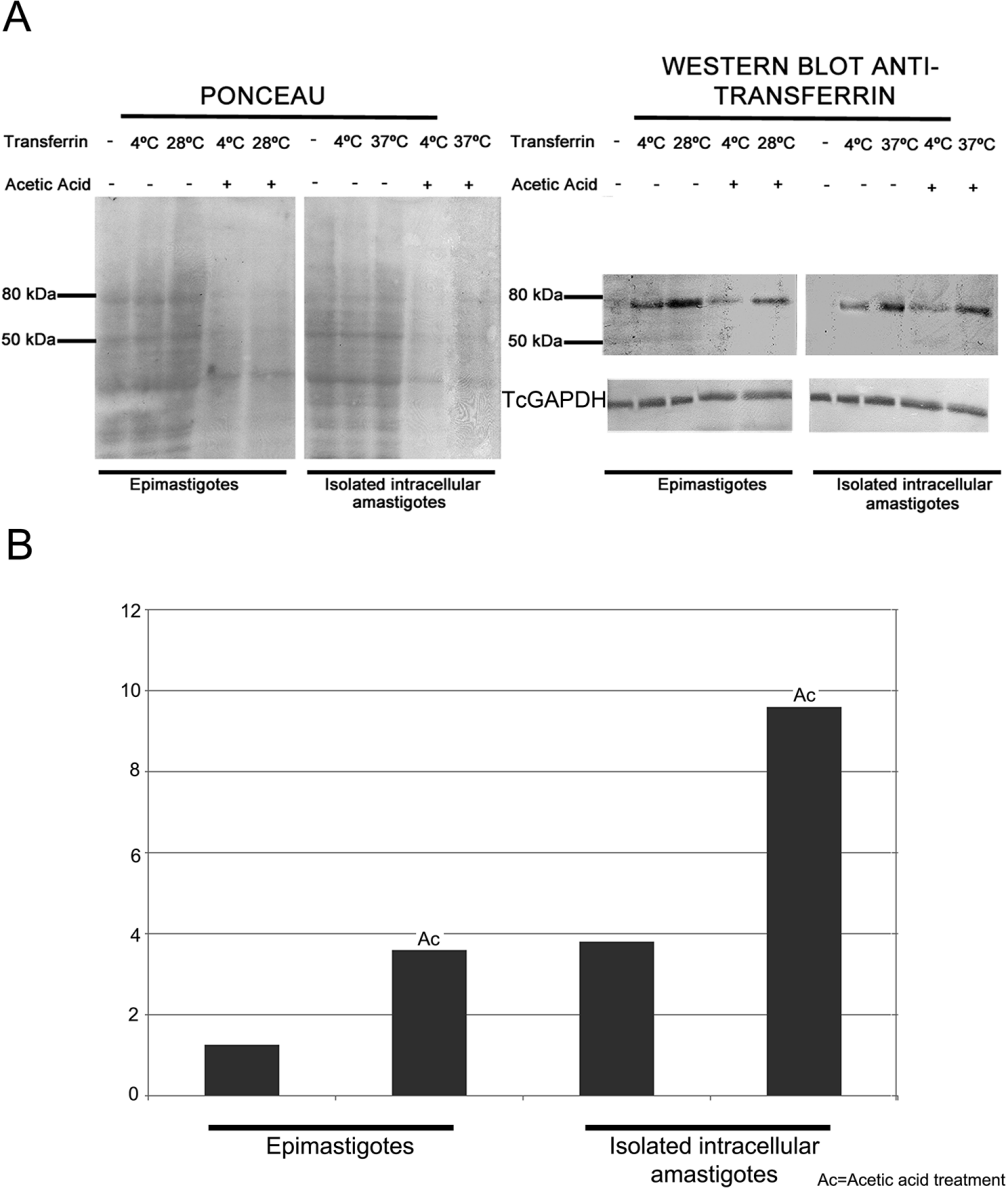
Western blotting analysis of transferrin-AlexaFluor 633 uptake in isolated *Trypanosoma cruzi* intracellular amastigotes. (A) Detection of holo-transferrin (using anti-transferrin goat antiserum) in total protein extracts of isolated intracellular amastigotes and epimastigotes (positive control for endocytosis) endocytosis assays at different temperatures. The panel on the left shows the Ponceau-stained membrane used for transferrin immuno-blotting analysis (on the right). The protein band with approximately 80 kDa corresponds to transferrin. Protein extract from isolated intracellular amastigotes incubated with transferrin at 37°C showed a strong band, indicating transferrin internalization. TcGAPDH (~40 kDa) was used as a loading control. (B) Ratios between the integrated densities of the transferrin band at 28 or 37°C and 4°C (Y-axis), based on measurements performed using ImageJ.

Using western blotting, we confirmed the presence of holo-transferrin (MW 76–81 kDa) in protein extracts of amastigotes and epimastigotes (positive control cells) subjected to a transferrin uptake assay (Fig 2). The anti-TcGAPDH antiserum used as a loading control recognized a polypeptide of the correct molecular weight (~40 kDa) in all *T*. *cruzi* developmental forms (Fig 2A).

Whole cell extracts of isolated intracellular amastigotes incubated at the endocytosis-permissive temperature of 37°C (likely to include both surface bound and internalized transferrin) contained more transferrin than extracts of parasites incubated at 4°C, when endocytosis does not occur and, thus, only surface-bound transferrin should be detected (Fig 2A). After a rapid (5s) acetic acid treatment, which leads to the dissociation of surface-bound transferrin [20], holo-transferrin levels in amastigotes incubated at 37°C remained similar to those observed without acetic acid treatment. In epimastigotes, transferrin localizes inside the flagellar pocket and the cytostome/cytopharynx complex, and then accumulates in the reservosomes [5,8]. While the intracellular pool of labeled transferrin is not accessible to acetic acid treatment, this treatment is expected to remove most surface-bound transferrin, in parasites incubated at 4°C. In this study, we observed a residual signal after acetic acid treatment in parasites incubated at 4°C (Fig 2A), probably due to surface-bound transferrin that could not be readily removed from to the interior of the cytostome/cytopharynx or from the flagellar pocket (Fig 2A). Densitometry analysis showed that the ratio between the transferrin signal at 28°C and that at 4°C was higher for parasites treated with acetic acid compared with untreated parasites (Fig 2B), in agreement with the hypothesis that acid treatment removed the predominantly surface-bound transferrin at 4°C, while the internalized transferrin prevalent in parasites incubated at 37°C was resistant to acid treatment.

Overall, the western blotting data suggest that transferrin binds to the surface of amastigotes (at 4 and 37°C) and is internalized by parasites (at 37°C). A positive control for endocytosis using epimastigotes (at an endocytosis-permissive temperature of 28°C, instead of 37°C) showed a very similar pattern to that observed for amastigotes, supporting our interpretation of the amastigote data.

### Isolated intracellular amastigotes have higher intracellular levels of labeled transferrin than axenic amastigotes

**Fig 3:**
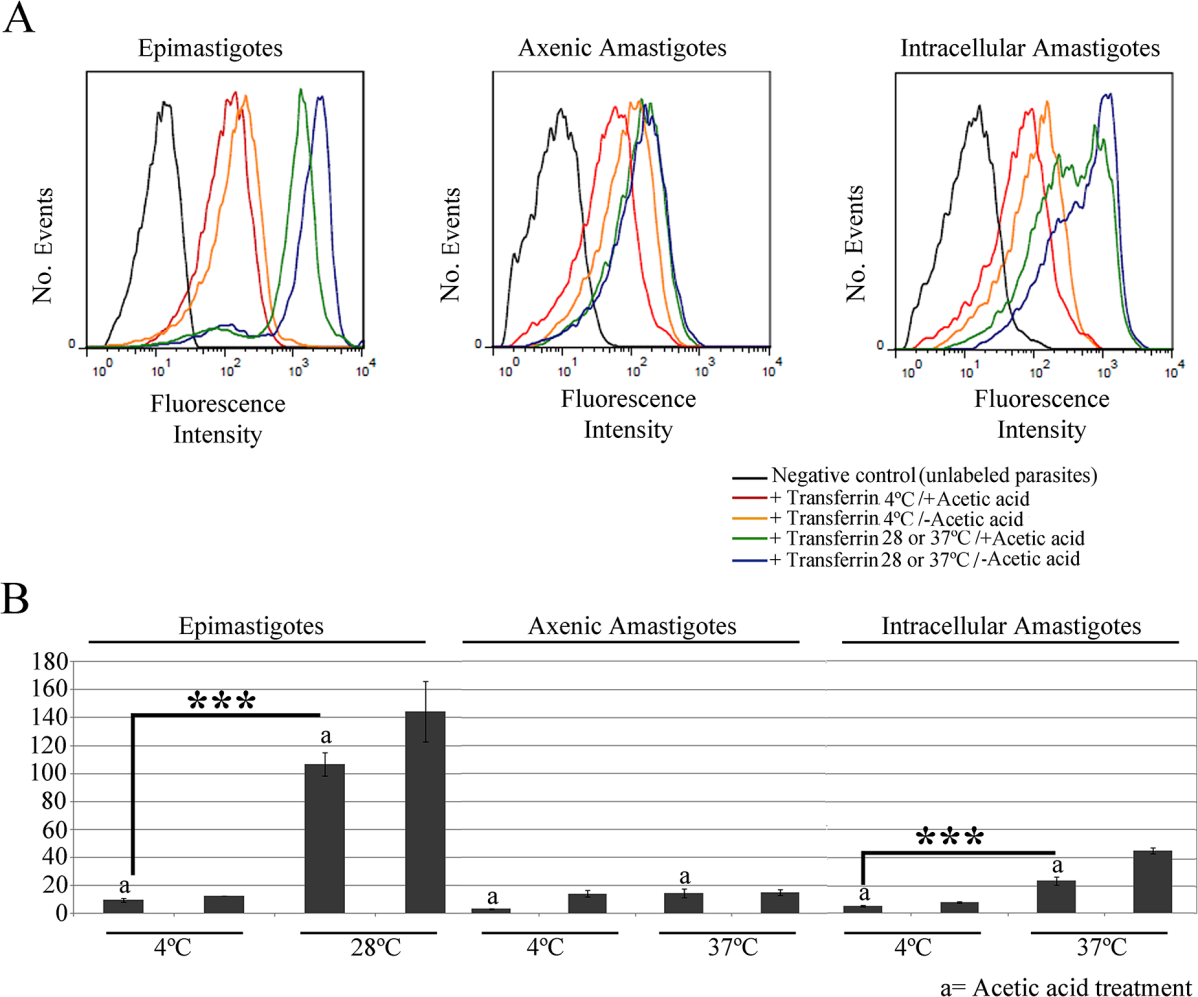
Flow cytometry analysis of transferrin-AlexaFluor 633 endocytosis by amastigotes and epimastigotes of *Trypanosoma cruzi*. (A) Flow cytometry histograms of parasites incubated with transferrin at different temperatures, and then treated with acetic acid, or left untreated. Epimastigotes were used as positive control, and negative control parasites (black) were incubated in medium without labeled transferrin. These data show that isolated intracellular amastigotes internalize transferrin at 37°C. (B) Normalized medians (stained/unstained control) of the fluorescence peaks. Note the low endocytosis levels in axenic amastigotes (N = 3). ***p<0.001. a, acetic acid treatment.

Flow cytometry analysis provided further support to the notion that transferrin is internalized by isolated intracellular amastigotes, since the transferrin-AlexaFluor 633 signal in these parasites increased significantly (p<0.001) when endocytic assays were performed at the endocytosis-permissive temperature 37°C, compared with samples incubated at 4°C (Fig 3A). The fluorescence signal increased in cells incubated at 4°C, compared with controls without transferrin, in line with the western blotting data that suggest that binding of transferrin to the surface of isolated intracellular amastigotes occurs at this temperature. The transferrin-AlexaFluor 633 signal decreased after acetic acid treatment in isolated intracellular amastigotes incubated at 4°C, and also in those incubated at 37°C. However, the fluorescence intensity of cells incubated at 37°C and treated with acetic acid remained higher than that of cells incubated at 4°C, but not subjected to acid treatment (Fig 3A). These results suggest that a considerable proportion of the labeled transferrin in cells incubated at 37°C was found in internal organelles and, thus, was resistant to acid treatment (Fig 3A).

Similar results were obtained for a positive control for endocytosis using epimastigotes (at an endocytosis-permissive temperature of 28°C, instead of 37°C): the fluorescence signal was higher in cells incubated at 28°C, when compared with those incubated at 4°C (Fig 3A). Also, acetic acid treatment decreased the fluorescence intensity in both cases, although a higher signal remained in cells incubated at 37°C, indicating transferrin internalization.

Interestingly, we observed a relatively low fluorescence signal in axenic amastigotes incubated at 37°C, similar to that seen in cells incubated at 4°C (Fig 3A). These data indicate that axenic amastigotes ingest only a limited amount of transferrin and, therefore, do not represent a reliable model for studies on *T*. *cruzi* endocytosis. Normalized medians of the fluorescence peaks showed that the endocytic activity is higher in epimastigotes than in isolated intracellular amastigotes, while axenic amastigotes showed only minimal endocytic activity (Fig 3B).

### Labeled transferrin co-localizes with cruzipain in lysosomal-related organelles (LROs) of isolated intracellular amastigotes

**Fig 4:**
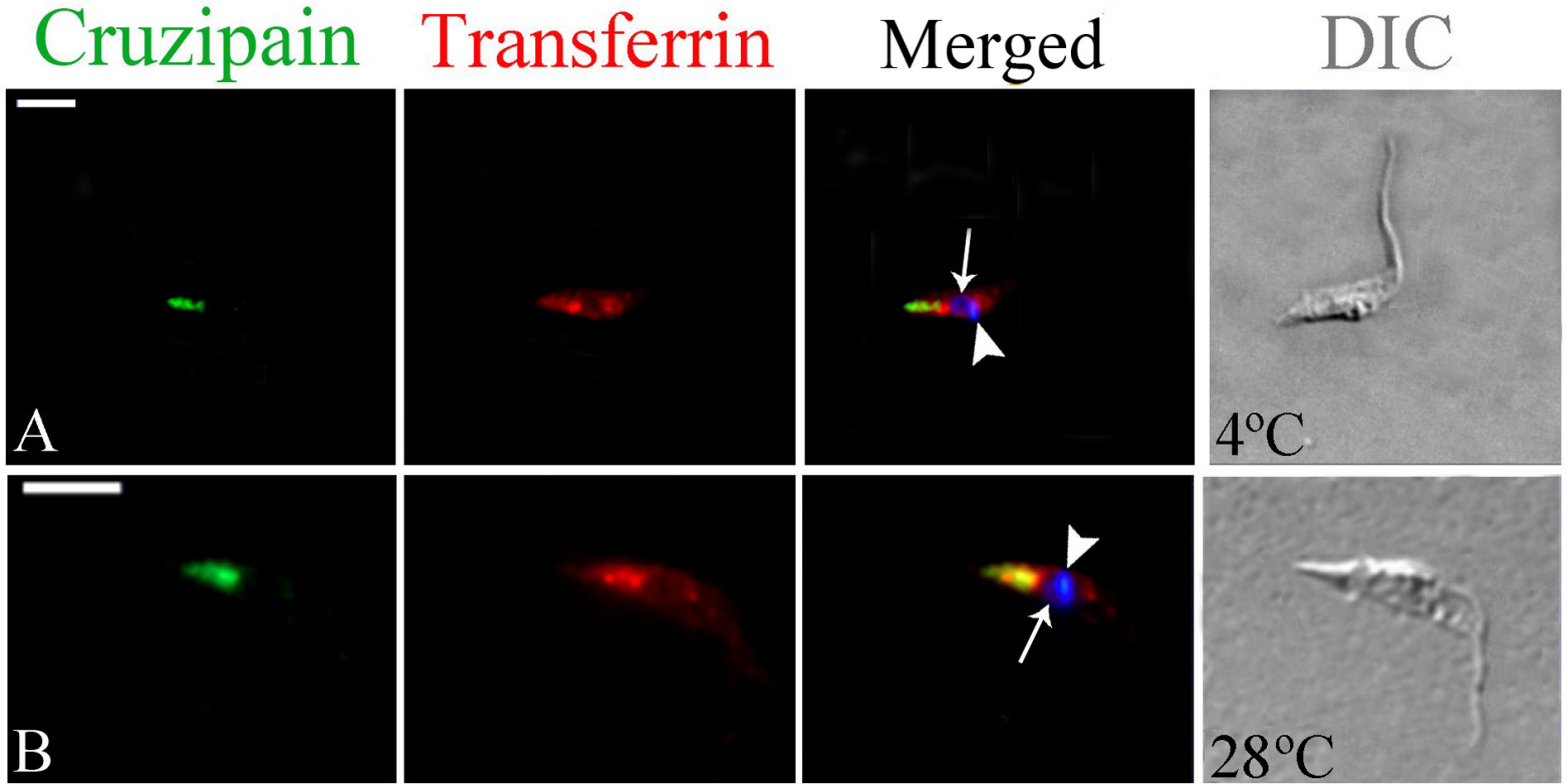
Fluorescence microscopy analysis of transferrin-AlexaFluor 633 endocytosis by *Trypanosoma cruzi* epimastigotes. Cells were allowed to ingest transferrin and were then labeled with an anti-transferrin antibody (in red) and with the anti-cruzipain mAb CZP-315.D9 (CZP; in green). At 4°C no co-localization was observed (A), while transferrin and cruzipain co-localized at the posterior region of the cell after incubation at 28°C (B), resulting in yellow staining of the reservosomes. The nucleus (arrow) and kinetoplast (arrowhead) are stained with Hoechst 33342 (in blue). DIC, differential interference contrast. Scale bars, 5 μm.

To examine the destination of the internalized transferrin, isolated intracellular amastigotes were subjected to endocytosis assays and then processed for immunofluorescence for the detection of transferrin and also of cruzipain, a marker of LROs (i.e., the reservosomes), the final destination of internalized transferrin in epimastigotes. In positive control cells (epimastigotes) incubated at 4°C, only cruzipain was detected in the reservosomes, located at the posterior end of the cell (Fig 4A and 4D), while transferrin was found at the anterior end of the cell (likely corresponding to the cytostome) and in dense spots close to the nucleus, likely corresponding to the bottom of the cytopharynx [9,17,44]. In epimastigotes incubated at 28°C there was co-localization of transferrin with cruzipain at the reservosomes (Fig 4E and 4H), as previously shown [11,12,39].

**Fig 5:**
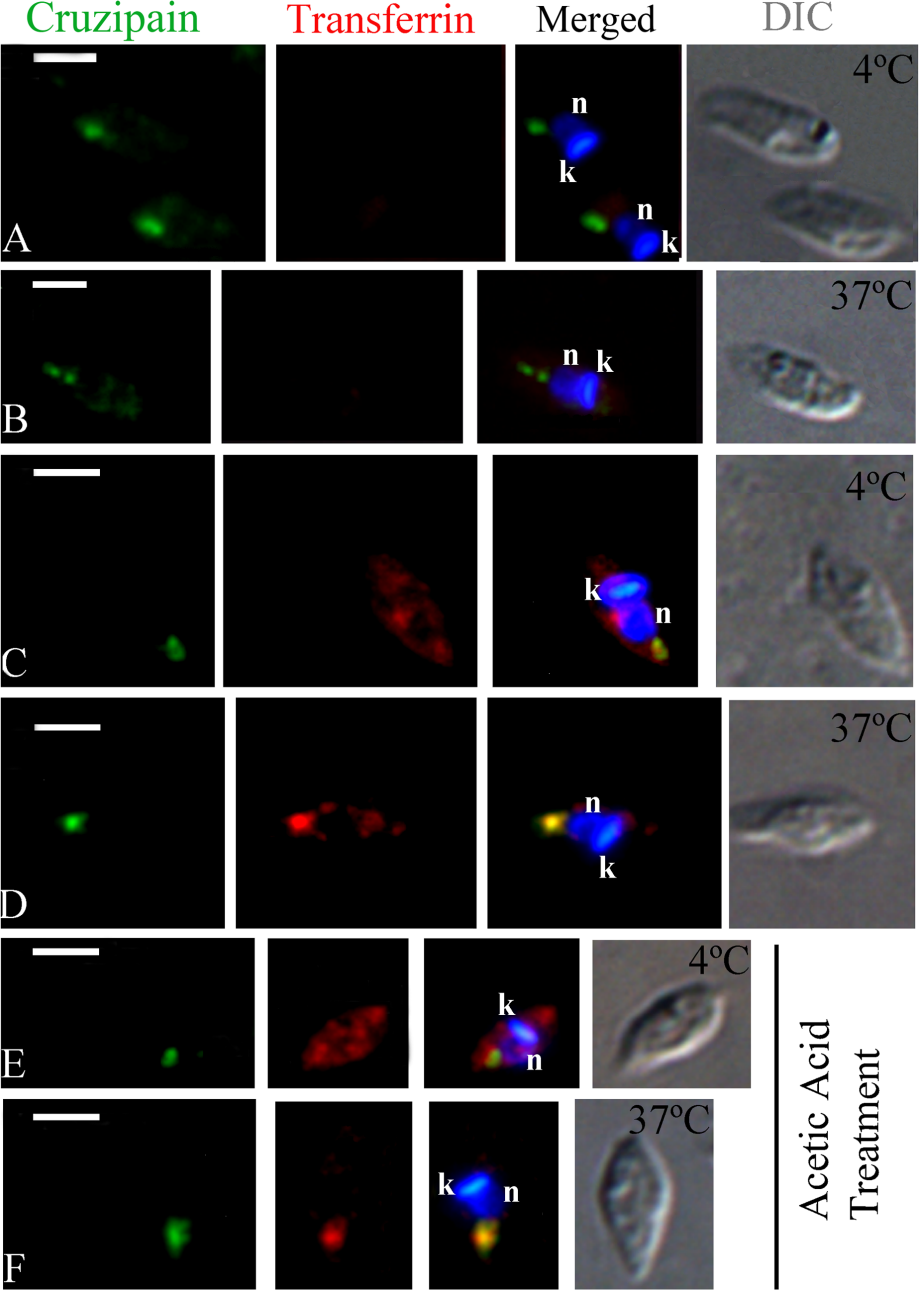
Fluorescence microscopy analysis of transferrin-AlexaFluor 633 endocytosis by isolated intracellular amastigotes of *Trypanosoma cruzi*. Cells were allowed to ingest transferrin-AlexaFluor 633 and were then incubated with anti-transferrin antibody (in red) and anti-cruzipain mAb (CZP-315.D9; in green). (A-B) Isolated intracellular amastigotes incubated without transferrin at 4°C or 37°C, showed no transferrin labeling (negative control), while cruzipain was located at the posterior region of the cell. (C) Isolated intracellular amastigotes incubated with transferrin at 4°C. No co-localization with cruzipain was observed. (D) Isolated intracellular amastigotes incubated with transferrin at 37°C. Co-localization with cruzipain was observed at the posterior region of the cell. (E-F) Isolated intracellular amastigotes incubated with transferrin at 4°C and 37°C and then subjected to acetic acid treatment. No co-localization of transferrin with cruzipain was observed at 4°C (E), while co-localization with cruzipain occurred at the posterior region at 37°C (F). The nucleus (n) and the kinetoplast (k) were stained with Hoechst 33342 (in blue). DIC, differential interference contrast. Scale bars, 2.5 μm.

As expected, transferrin labeling in isolated intracellular amastigotes (4 or 37°C) was not intense, but was clearly detectable in most cells. No co-localization with cruzipain occurred at 4°C: transferrin labeling was prominent at a discrete spot close to the nucleus and the kinetoplast (corresponding to the location of the bottom of the cytopharynx), while cruzipain localized to the posterior region of the cell (Fig 5A and 5B). In contrast, we detected co-localization of transferrin and cruzipain in LROs at the posterior region of the cell, in amastigotes incubated at 37°C, although co-localization was not clear in all cells (Fig 5C and 5D). A similar result was obtained when amastigotes were subjected to endocytosis assays and then treated with acetic acid (Fig 5E and 5F).These data indicate a low level of protein uptake; nevertheless, internalized transferrin was delivered to (and stored in) cruzipain-positive LROs at the posterior of the cell.

### Ingested albumin co-localizes with cruzipain in isolated intracellular amastigotes, confirming a storage function for amastigote LROs

**Fig 6:**
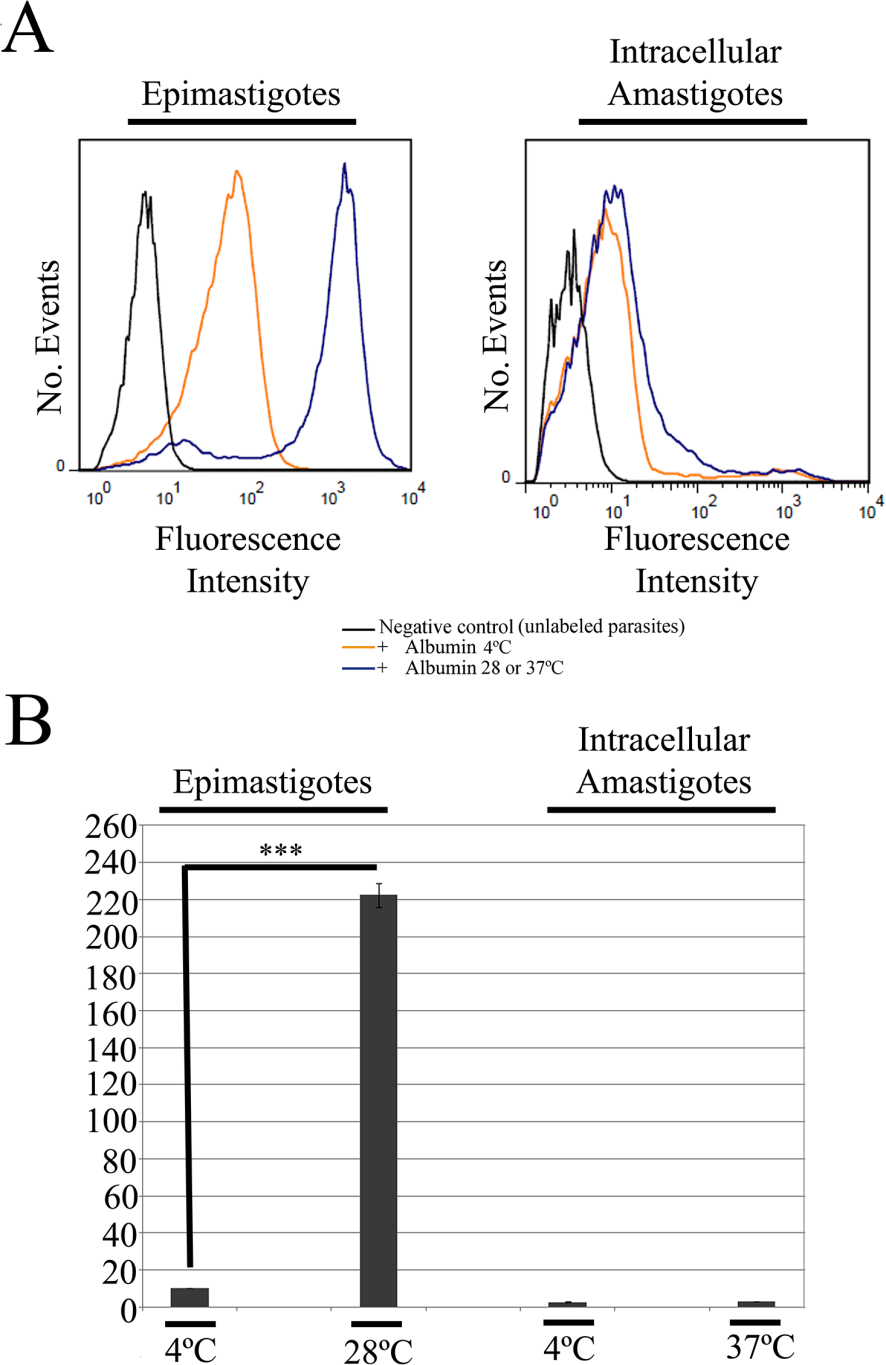
Flow cytometry analysis of albumin-AlexaFluor 488 endocytosis in *T*. *cruzi* by isolated intracellular amastigotes. (A) Flow cytometry histograms of cells incubated with labeled albumin at different temperatures. Epimastigotes were used as positive control, and negative control parasites (black) were incubated in medium without labeled albumin. These data show that isolated intracellular amastigotes internalize low amounts of albumin at 37°C. (B) Normalized medians of the fluorescence peaks, relative to the negative control (N = 3). ***p<0.001.

To examine the internalization of a different endocytic marker, we analyzed the uptake of albumin-AlexaFluor 488 complexes by isolated intracellular amastigotes, using flow cytometry. As expected, we observed an intense fluorescence signal indicative of albumin internalization in epimastigotes (i.e., positive control cells) incubated with Albumin-AlexaFluor 488 at 28°C; this signal was significantly higher (p<0.001) than that observed in controls cells incubated at 4°C or not given the labeled tracer (Fig 6). However, only a weak albumin-AlexaFluor 488 fluorescence signal was detected in isolated intracellular amastigotes, regardless the experimental condition (Fig 6), indicating minimal endocytosis of albumin by this developmental form.

**Fig 7:**
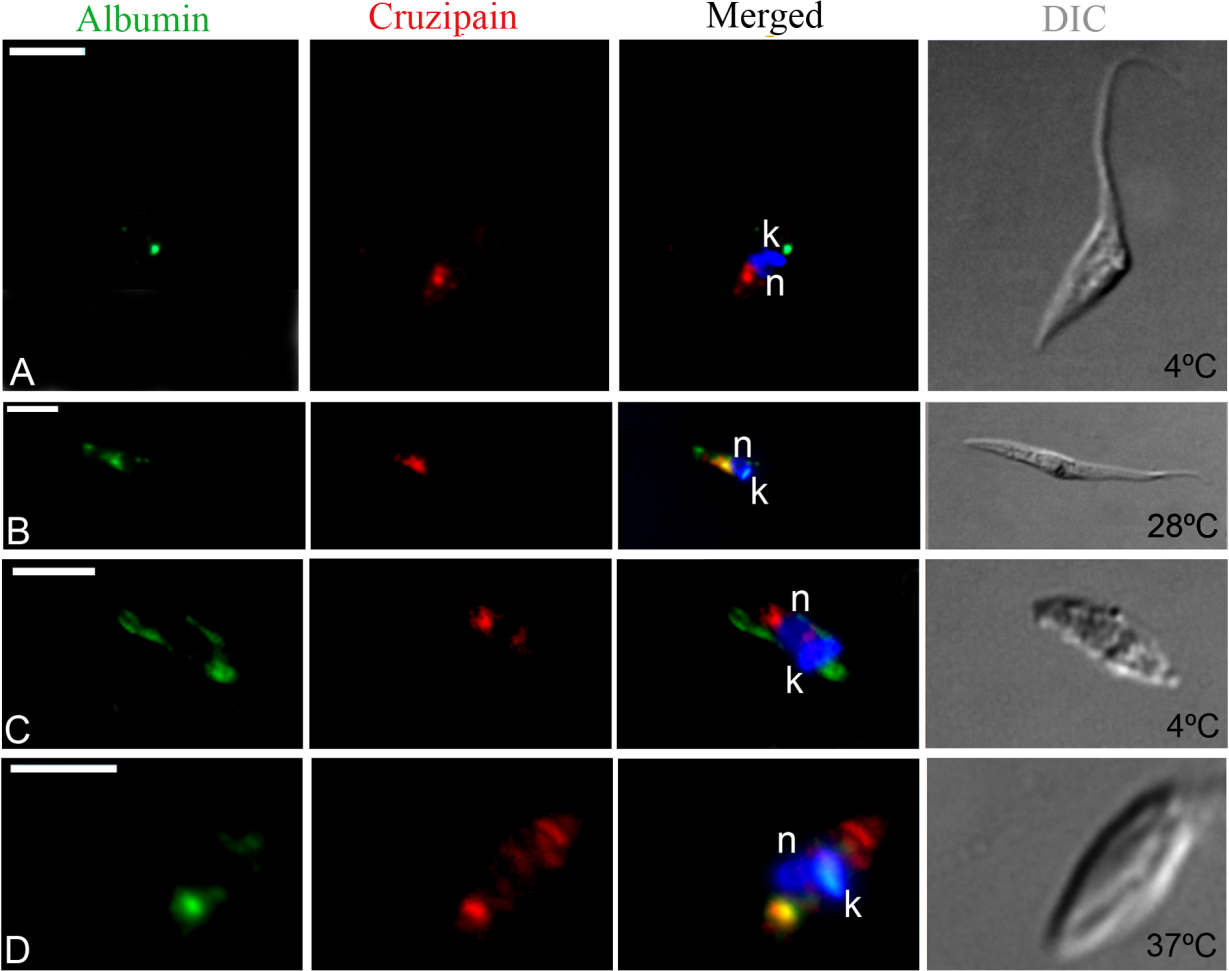
Fluorescence microscopy analysis of albumin-AlexaFluor 488 endocytosis by isolated intracellular amastigotes of *T*. *cruzi*. Cells were allowed to ingest albumin-AlexaFluor 488 (in green) and were then labeled with the anti-cruzipain mAb CZP-315.D9 (in red). In epimastigotes (A) and isolated intracellular amastigotes (C) incubated at 4°C, we did not observe co-localization of albumin (at the anterior end of the cell, nearest to the kinetoplast—k) and cruzipain (at the posterior end of the cell, nearest to the nucleus—n). In contrast, in epimastigotes (B) and isolated intracellular amastigotes (D) incubated at endocytosis-permissive temperatures (28°C or 37°C, respectively), we detected co-localization of albumin and cruzipain at the posterior region of the cell. The nucleus (n) and kinetoplast (k) are stained with Hoechst 33342 (in blue). DIC, differential interference contrast. Scale bars, 5 μm.

Despite the failure to detect albumin-AlexaFluor 488 internalization by flow cytometry, we could detect endocytosis of this labeled tracer by isolated intracellular amastigotes using fluorescence microscopy (Fig 7); however, labeling was relatively weak and not observed in all cells. Control experiments with epimastigotes showed that, at 4°C, albumin-AlexaFluor 488 complexes were occasionally found at the flagellar pocket region, but did not co-localize with cruzipain in the reservosomes (Fig 7A). However, at 28°C albumin and cruzipain co-localized in reservosomes, as shown here for the first time (Fig 7B). Similar results were obtained with isolated intracellular amastigotes: at 4°C no co-localization was observed (Fig 7C), while at 37°C albumin and cruzipain occasionally co-localized at LROs (Fig 7D). At both temperatures cruzipain was also detected in the anterior region of cell, maybe *en route* to LROs and/or to the secretory pathway. These results, combined with the flow cytometry data, indicate that amastigotes are capable of albumin uptake, albeit only at low levels.

**Fig 8:**
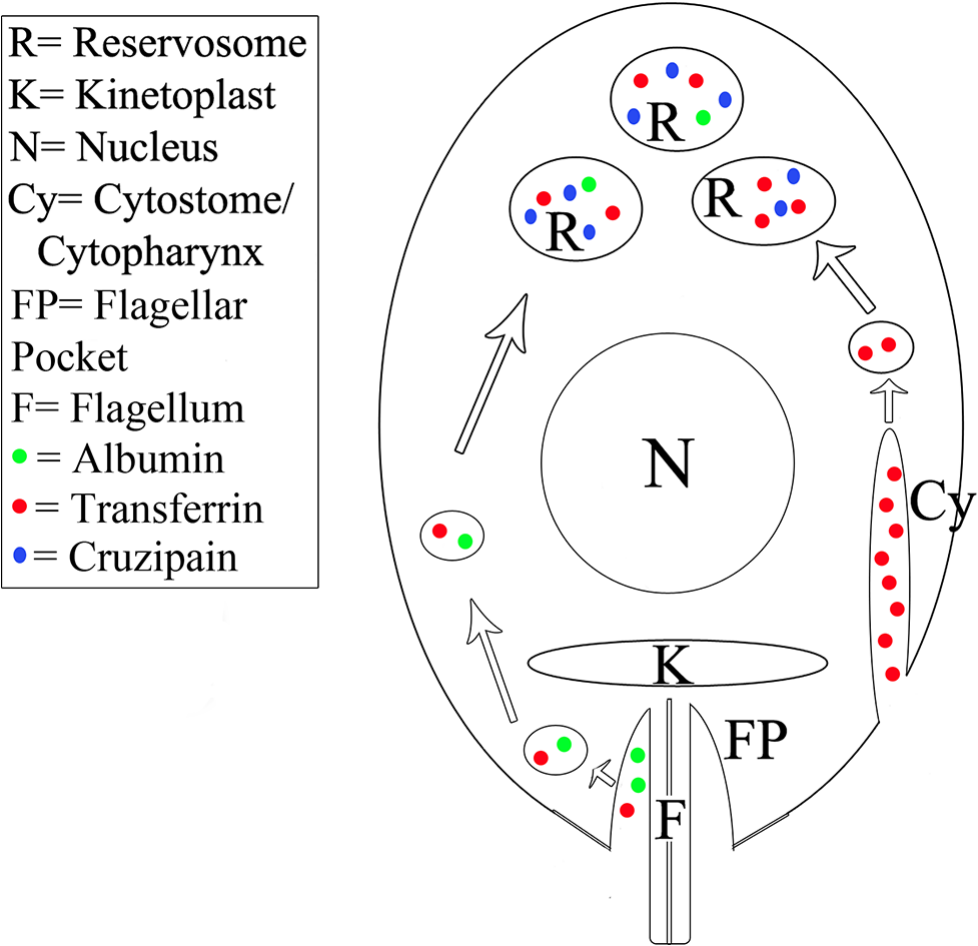
Schematic model of transferrin and albumin uptake by isolated *Trypanosoma cruzi* intracellular amastigotes. As in epimastigotes, transferrin uptake by amastigotes occurs mainly through the cytostome/cytopharynx complex (Cy). After internalization, transferrin is directed to the reservosomes (R), where it is stored together with lysosomal proteases such as cruzipain (Cz), the major cysteine protease of this parasite. Albumin (A) is internalized via the flagellar pocket region, but albumin endocytic activity is low. The destination of ingested macromolecules is the reservosomes.

Thus, contrary to the suggestion that amastigote LROs are not *bona fide* components of the endocytic pathway [13], our findings show that *T*. *cruzi* amastigote forms internalize macromolecules and target ingested cargo to cruzipain-positive LROs. To our knowledge, these data represent the first direct evidence that amastigote LROs correspond to the reservosomes observed in epimastigote forms (Fig 8).

## Conclusions

Host cell lysis by nitrogen decompression (cavitation) allows rapid and efficient isolation of viable *T*. *cruzi* intracellular amastigotes that have the typical amastigote shape and are positive for the amastigote-specific antigen Ssp4. Our data show that isolated intracellular amastigotes of *T*. *cruzi* display endocytic activity, although at lower levels than epimastigotes. Also, we show that internalized cargo (transferrin and albumin) accumulates in amastigote LROs, a clear indication that these organelles are functionally related to the reservosomes observed in epimastigotes.

## Supporting Information

S1 Fig Flow cytometry analysis of intracellular amastigotes release by nitrogen cavitation. Density plots showing the distribution of cell populations before and after Vero cell lysis by nitrogen decompression at 180 psi, compared with the profile obtained after trypsinization, but before nitrogen decompression. Vero cell cultures were infected with a GFP-expressing (GFP+) *T*. *cruzi* cell line, to verify host cell disruption and amastigote (Ama) release. Almost all infected Vero cells were disrupted after cavitation, releasing GFP+ amastigotes. Cell gates are marked in the plot on the right.
(TIF)

## References

[1] Mayor S, Pagano RE. Pathways of clathrin-independent endocytosis. Nat Rev Mol Cell Biol. 2007; 8: 603–612. 17609668 10.1038/nrm2216 PMC7617177

[2] Lundmark R, Carlsson SR. Driving membrane curvature in clathrin-dependent and clathrin-independent endocytosis. Sem Cell Develop Biol. 2010; 21: 363–370. 10.1016/j.semcdb.2009.11.014 19931628

[3] Mayor S, Parton RG, Donaldson JG. Clathrin-independent pathways of endocytosis. Cold Spring Harb Perspect Biol. 2014; 6: a016758 10.1101/cshperspect.a016758 24890511 PMC4031960

[4] Kirchhausen T, Owen D, Harrison SC. Molecular structure, function, and dynamics of clathrin-mediated membrane traffic. Cold Spring Harb Perspect Biol. 2014; 6: a016725 10.1101/cshperspect.a016725 24789820 PMC3996469

[5] Soares MJ, De Souza W. Endocytosis of gold-labeled proteins and LDL by *Trypanosoma cruzi* . Parasitol Res. 1991; 77: 461–468. 1656428 10.1007/BF00928410

[6] Figueiredo RC, Soares MJ. Low temperature blocks fluid-phase pinocytosis and receptor-mediated endocytosis in *Trypanosoma cruzi* epimastigotes. Parasitol Res. 2000; 86: 413–418. 10836515 10.1007/s004360050686

[7] Kalb LC, Frederico YCA, Batista CM, Eger I, Fragoso SP, Soares MJ. Clathrin expression in *Trypanosoma cruzi* . BMC Cell Biol. 2014; 15: 23 10.1186/1471-2121-15-23 24947310 PMC4073184

[8] De Souza W, Sant´Anna C, Cunha-e-Silva NL. Electron microscopy and cytochemistry analysis of the endocytic pathway of pathogenic protozoa. Prog Histochem Cytochem. 2009; 44: 67–124. 10.1016/j.proghi.2009.01.001 19410686

[9] Alcantara CL, Vidal JC, de Souza W, Cunha-e-Silva NL. The three-dimensional structure of the cytostome-cytopharynx of *Trypanosoma cruzi* epimastigotes. J Cell Sci. 2014; 127: 2227–2237. 10.1242/jcs.135491 24610945

[10] Porto-Carreiro I, Attias M, Miranda K, De Souza W, Cunha-e-Silva NL. *Trypanosoma cruzi* epimastigote endocytic pathway: cargo enters the cytostome and passes through an early endosomal network before storage in reservosomes. Eur J Cell. 2000; Biol 79: 858–69. 11139150 10.1078/0171-9335-00112

[11] Soares MJ, Souto-Padrón T, De Souza W. Identification of a large pre-lysosomal compartment in the pathogenic protozoon *Trypanosoma cruzi* . J Cell Sci. 1992; 2: 157–167. 10.1242/jcs.102.1.157 1500438

[12] Cazzulo JJ, Cazzulo Franke MC, Martinez J, Franke de Cazzulo BM. Some kinetic properties of a cysteine proteinase (cruzipain) from *Trypanosoma cruzi* . Biochem Biophys Acta. 1990; 1037: 186–191. 2407295 10.1016/0167-4838(90)90166-d

[13] Sant’Anna C, Parussini F, Lourenço D, De Souza W, Cazzulo JJ, Cunha-e-Silva NL. All *Trypanosoma cruzi* developmental forms present lysosome-related organelles. Histochem Cell Biol. 2008; 130: 1187–1198. 10.1007/s00418-008-0486-8 18696100

[14] Bothwell H, Charlton RW, Cook JD, Finch CH. Iron metabolism in man Oxford: Blackwell; 1979.

[15] Taylor MC, Kelly JM. Iron metabolism in trypanosomatids, and its crucial role in infection. Parasitol. 2010; 6: 899–917. 10.1017/S0031182009991880 20152063

[16] Corrêa JR, Atella GC, Batista MM, Soares MJ. Transferrin uptake in *Trypanosoma cruzi* is impaired by interference on cytostome-associated cytoskeleton elements and stability of membrane cholesterol, but not by obstruction of clathrin-dependent endocytosis. Exp Parasitol. 2008; 119: 58–66. 10.1016/j.exppara.2007.12.010 18234197

[17] Milder R, Deane MP. The cytostome of *Trypanosoma cruzi* and *T*. *conorhini* . J Protozool. 1969; 16: 730–737. 5362390 10.1111/j.1550-7408.1969.tb02335.x

[18] Sant’Anna C, Pereira MG, Lemgruber L, De Souza W, Cunha-e-Silva NL. New insights into the morphology of *Trypanosoma cruzi* reservosome. Microsc Res Tech. 2008; 71: 599–605. 10.1002/jemt.20592 18452191

[19] Meyer H, De Souza W. On the fine structure of *Trypanosoma cruzi* in tissue culture of pigment epithelium from the chick embryo. Uptake of melanin granules by the parasite. J Protozool. 1973; 20: 590–593. 4128532 10.1111/j.1550-7408.1973.tb03580.x

[20] Lima MF, Villalta F. *Trypanosoma cruzi* receptors for human transferrin and their role. Mol Biochem Parasitol. 1990; 38: 245–252. 2183049 10.1016/0166-6851(90)90027-j

[21] Loo VG, Lalonde RG. Role of iron in intracellular growth of *Trypanosoma cruzi* . Infect Immun. 1984; 45: 726–730. 6381312 10.1128/iai.45.3.726-730.1984 PMC263357

[22] Lalonde RG, Holbein BE. Role of iron in *Trypanosoma cruzi* infection in mice. J Clin Invest. 1984; 73: 470–476. 6421877 10.1172/JCI111233 PMC425038

[23] Waghabi MC, Keramidas M, Bailly S, Degrave W, Mendonça-Lima L, Soeiro MN, et al Uptake of host cell transforming growth factor-β by *Trypanosoma cruzi* amastigotes in cardiomyocytes: potential role in parasite cycle completion. Am J Pathol. 2005; 167: 993–1003. 16192635 10.1016/s0002-9440(10)61189-3 PMC1603686

[24] Mayle KM, Le AM, Kamei D. The intracellular trafficking pathway of transferrin. Biochim Biophis Acta. 2012; 1820: 264–281. 10.1016/j.bbagen.2011.09.009 21968002 PMC3288267

[25] Abrahamsohn IA, Katzin AM, Milder RV. A method for isolating *Trypanosoma cruzi* amastigotes from spleen and liver using two-step discontinuous gradient centrifugation. J Parasitol. 1983; 69: 437–439. 6343578

[26] Villalta F, Kierszenbaum F. Growth of isolated amastigotes of *Trypanosoma cruzi* in cell-free medium. J Protozool. 1982; 29: 570–576. 6816925 10.1111/j.1550-7408.1982.tb01338.x

[27] de Carvalho TU, De Souza W. Separation of amastigotes and trypomastigotes of *Trypanosoma cruzi* from cultured cells. Z Parasitenkd. 1983; 69: 571–575. 6356671 10.1007/BF00926668

[28] Marques AF, Nakayasu ES, Almeida IC. Purification of extracellular and intracellular amastigotes of *Trypanosoma cruzi* from mammalian host-infected cells. Protocol Exchange. 2011; 10.1038/protex.2011.265

[29] de Sousa MA. A simple method to purify biologically and antigenically preserved bloodstream trypomastigotes of *Trypanosoma cruzi* using DEAE-cellulose columns. Mem Inst Oswaldo Cruz. 1983; 78: 317–333. 6361445 10.1590/s0074-02761983000300009

[30] Contreras VT, Araque W, Delgado VS. *Trypanosoma cruzi*: metacyclogenesis *in vitro*—I. Changes in the properties of metacyclic trypomastigotes maintained in the laboratory by different methods. Mem Inst Oswaldo Cruz. 1994; 89: 253–259. 7885254 10.1590/s0074-02761994000200026

[31] Contreras VT, Araújo-Jorge TC, Bonaldo MC, Thomaz N, Barbosa HS, Goldenberg S. Biological aspects of the DM28c clone of *Trypanosoma cruzi* after metacyclogenesis in chemically defined media. Mem Inst Oswaldo Cruz. 1988; 83: 123–133. 3074237 10.1590/s0074-02761988000100016

[32] Camargo EP. Growth and differentiation in *Trypanosoma cruzi*. I. Origin of metacyclic trypanosomes in liquid media. Rev Inst Med Trop São Paulo. 1964; 6: 93–100. 14177814

[33] Contreras VT, Salles JM, Thomas N, Morel CM, Goldenberg S. *In vitro* differentiation of *Trypanosoma cruzi* under chemically defined conditions. Mol Biochem Parasitol. 1985; 16: 315–327. 3903496 10.1016/0166-6851(85)90073-8

[34] Hernándes-Osorio LA, Marquez-Dulñas C, Florencio-Martínez LE, Ballesteros-Rodea G, Martínez-Calvillo S, Manning-Cela RG. Improved method for i*n vitro* secondary amastigogenesis of *Trypanosoma cruzi*: morphometrical and molecular analysis of intermediate developmental forms. J Biomed Biotech. 2010: Article ID 283842, 10 pages. 10.1155/2010/283842 PMC2796335 20037731

[35] Sambrook J, Fritsch EF, Maniatis T. Detection and analysis of proteins expressed from cloned genes In Molecular cloning: A laboratory manual. Vol. 3, 2nd edition Woodbury: Cold Spring Harbor Laboratory Press; 1989 pp. 60–74.

[36] Gradia DF, Rau K, Umaki AC, de Souza FS, Probst CM, Correa A, et al Characterization of a novel Obg-like ATPase in the protozoan *Trypanosoma cruzi* . Int J Parasitol. 2009; 39: 49–58. 10.1016/j.ijpara.2008.05.019 18713637

[37] Kessler RL, Soares MJ, Probst CM, Krieger MA. *Trypanosoma cruzi* response to sterol biosynthesis inhibitors: morphophysiological alterations leading to cell death. PLOS ONE. 2013; 8(1): e55497 10.1371/journal.pone.0055497 23383204 PMC3561218

[38] Andrews NW, Hong KS, Robbins ES, Nussenzweig V. Stage-specific surface antigens expressed during the morphogenesis of vertebrate forms of *Trypanosoma cruzi* . Exp Parasitol. 1987; 64: 474–484. 3315736 10.1016/0014-4894(87)90062-2

[39] Batista CM, Medeiros LC, Eger I, Soares MJ. mAb CZP-315.D9: an anti-recombinant cruzipain monoclonal antibody that specifically labels the reservosomes of *Trypanosoma cruzi* epimastigotes. Biomed Res Int. 2014; Article ID 714749, 9 pages. 10.1155/2014/714749 PMC3920967 24587988

[40] Urbina JA. Specific chemotherapy of Chagas disease: relevance, current limitations and new approaches. Acta Tropica. 2010; 115: 55–68. 10.1016/j.actatropica.2009.10.023 19900395

[41] Simpson RJ. Disruption of cultured cells by nitrogen cavitation. Cold Spring Harb Protoc. 2010; 10.1101/pdb.prot5513 21041386

[42] De Souza W. Electron microscopy of trypanosome- A historical view. Mem Inst Oswaldo Cruz. 2008; 103: 313–325. 18660983 10.1590/s0074-02762008000400001

[43] Kessler RL, Gradia DF, Pontello Rampazzo R de C, Lourenço ÉE, Fidêncio NJ, Manhaes L, et al Stage-regulated GFP Expression in *Trypanosoma cruzi*: applications from host-parasite interactions to drug screening. PLOS ONE. 2013; 8: e67441 10.1371/journal.pone.0067441 23840703 PMC3688654

[44] Ramos TC, Freymüller-Haapalainen E, Schenkman S. Three-dimensional reconstruction of *Trypanosoma cruzi* epimastigotes and organelle distribution along the cell division cycle. Cytometry A. 2011; 79: 538–544. 10.1002/cyto.a.21077 21567937

