# Supplementary figures and images for: Trypanosoma cruzi Intracellular Amastigotes Isolated by Nitrogen Decompression Are Capable of Endocytosis and Cargo Storage in Reservosomes
Source: PLoS One. 2015 Jun 9;10(6):e0130165. doi: 10.1371/journal.pone.0130165 (PMC4461355; doi:10.1371/journal.pone.0130165)

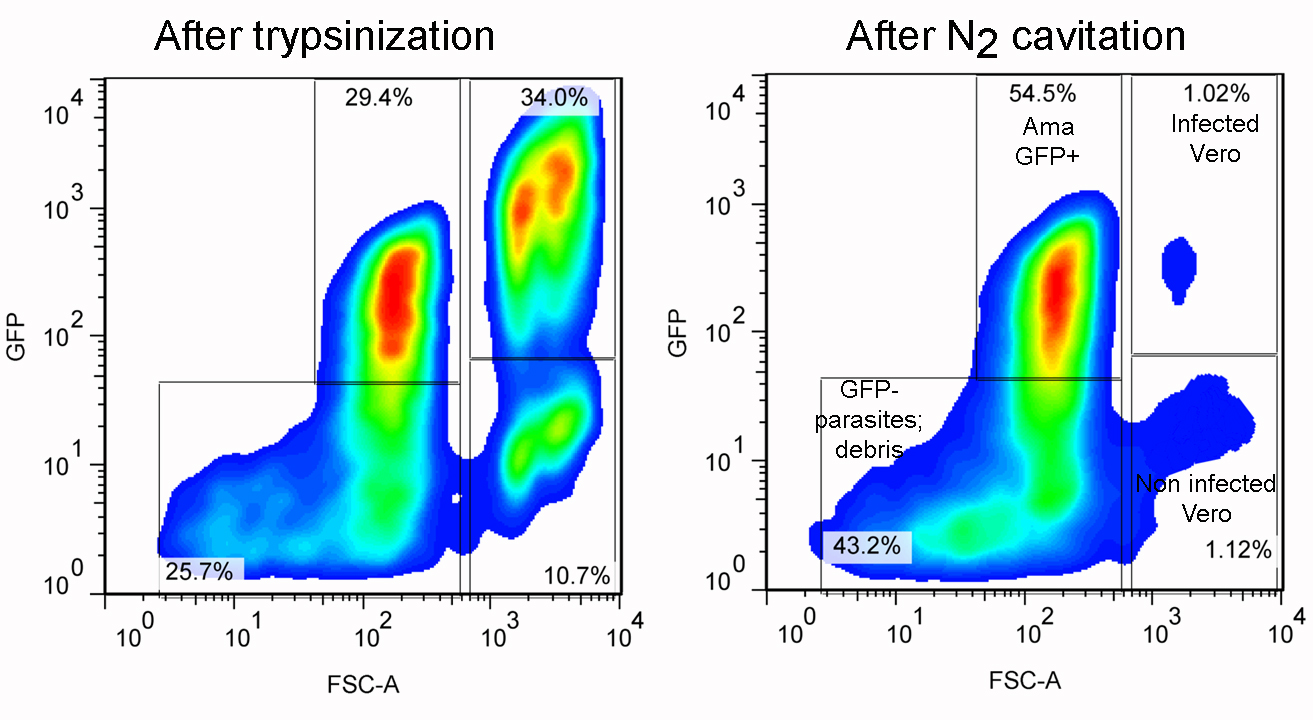

Supplement: S1 Fig — Density plots showing the distribution of cell populations before and after Vero cell lysis by nitrogen decompression at 180 psi, compared with the profile obtained after trypsinization, but before nitrogen decompression. Vero cell cultures were infected with a GFP-expressing (GFP+) T. cruzi cell line, to verify host cell disruption and amastigote (Ama) release. Almost all infected Vero cells were disrupted after cavitation, releasing GFP+ amastigotes. Cell gates are marked in the plot on the right. (TIF) [file pone.0130165.s001.tif]
